# Death by SARS-CoV 2: a Romanian COVID-19 multi-centre comorbidity study

**DOI:** 10.1038/s41598-020-78575-w

**Published:** 2020-12-10

**Authors:** Anca Pantea Stoian, Mihaela Pricop-Jeckstadt, Adrian Pana, Bogdan-Vasile Ileanu, Ruxandra Schitea, Marius Geanta, Doina Catrinoiu, Andra Iulia Suceveanu, Cristian Serafinceanu, Silviu Pituru, Catalina Poiana, Bogdan Timar, Cornelia Nitipir, Simona Parvu, Andreea Arsene, Laura Mazilu, Antonela Toma, Razvan Hainarosie, Antonio Ceriello, Manfredi Rizzo, Viorel Jinga

**Affiliations:** 1 University of Medicine and Pharmacy “Carol Davila”, 37 Dionisie Lupu Str., 020021 Bucharest, Romania; 2 National Institute of Diabetes, Nutrition and Metabolic Diseases “N. C. Paulescu”, 5-7. Ion Movila Str, 030167 Bucharest, Romania; 3 Department of Applied Mathematics, University POLITEHNICA of Bucharest, Splaiul Independentei 313, 060042 Bucharest, Romania; 4 Center for Research and Training in Innovative Techniques of Applied Mathematics in Engineering-Traian Lalescu-(CiTi), University Politehnica of Bucharest, Splaiul Independentei 313, 060042 Bucharest, Romania; 5 Center for Health Outcomes & Evaluation, Splaiul Unirii 45, 030126 Bucharest, Romania; 6 Center for Innovation in Medicine, Bd. Theodor Pallady, No. 42J, Room 1719, 032266 Bucharest, Romania; 7 Department of Internal Medicine, Faculty of Medicine, Ovidius University of Constanta, University C2 Street, 900527 Constanţa, Romania; 8 Victor Babes” University of Medicine and Pharmacy, 2 Eftimie Murgu, 300041 Timisoara, Romania; 9 Department of Mathematical Methods and Models, University POLITEHNICA of Bucharest, Splaiul Independentei 313, 060042 Bucharest, Romania; 10 Prof. Dr. D. Hociota” Institute of Phonoaudiology and Functional ENT Surgery, 21st Mihail Cioranu Street, 061344 Bucharest, Romania; 11 IRCCS MultiMedica, 300 Milanese Str, 20099 Milan, Sesto San Giovanni Italy; 12 Division of Endocrinology, Diabetes and Metabolism, Department of Medicine, University of South Carolina, 6311 Garners Ferry Rd, Columbia, SC 29209 USA; 13 Department of Health Promotion, Mother and Child Care, Internal Medicine and Medical Specialties, University of Palermo, 61 Piazza Marina Str, 90133 Palermo, Italy

**Keywords:** Diseases, Medical research, Risk factors

## Abstract

Evidence regarding the relation between SARS-CoV-2 mortality and the underlying medical condition is scarce. We conducted an observational, retrospective study based on Romanian official data about location, age, gender and comorbidities for COVID-19 fatalities. Our findings indicate that males, hypertension, diabetes, obesity and chronic kidney disease were most frequent in the COVID-19 fatalities, that the burden of disease was low, and that the prognosis for 1-year survival probability was high in the sample. Evidence shows that age-dependent pairs of comorbidities could be a negative prognosis factor for the severity of disease for the SARS-CoV 2 infection.

## Introduction

The rapid spread of the new type of coronavirus named novel Severe Acute Respiratory Syndrome Coronavirus 2 (SARS-COV-2) from Wuhan (China) in the world determined the World Health Organization (WHO) to declare novel coronavirus disease (2019 COVID-19 or nCOVID-19) a pandemic on March 11, 2020^1,2^.

The first confirmed case in Romania was on February 26, 2020, and, by April 20 2020, 8936 confirmed case, 2017 healed cases and 520 deaths had been recorded, 88 cases with missing data and 98,491 tests were done^3^. The case definitions, as well as the therapies applied by the Romanian physicians, were also used in Italy, Spain and France^4–7^. The diagnostic was established based on the clinical symptomatology like cough, temperature, breathing difficulty without a prescribed etiology, on patient's direct exposure to SARS-COV-2, and the corroboration by testing with the RT-PCR method^8,9^.

The Romanian government took strict measures to limit the outbreak, and a Coronavirus task force was set up to coordinate this effort under the direct supervision of the Ministry of Health (MS). Even though some cases of intra-hospital or community infection arose, they were isolated and contained with the help of special hygiene measures, social distancing and a very tight lockdown imposed by the authorities. However, a large number of deaths were confirmed among the adult population with no patient under 20 years of age dying because of COVID-19. Hence, issues regarding the COVID-19 mortality, especially the relationship with the comorbidities and the burden of disease are of great importance for the medical community^8^.

Several studies about the comorbidities of the patients infected with SARS-COV-2 indicate hypertension, diabetes, obesity, neoplasms, chronic kidney disease and chronic obstructive pulmonary disease (COPD) as leading risk factors for a lethal evolution of the disease^10–12^. Romania holds a top place in Europe for deaths caused by cardiovascular diseases with hypertension affecting over 45% of the adult population. Almost half of the population aged over 65 has at least one chronic disease, and 1 in 5 adults smokes daily (32% men as compared to 18% women)^13^. Moreover, Romania has a record number of deaths due to preventable causes like ischemic cardiac disease, pulmonary cancer or alcoholism^14^. The prevalence of diabetes is approximately 11.6% and doubles for prediabetes as confirmed by Predatorr study^15^ and Mentor study^16^. Therefore, it is expected that a high number of infections with SARS-CoV-2 in Romania to have a severe course of the disease or to end deadly.

To investigate the comorbidity profiles for SARS-CoV2 deaths, an observational retrospective study was conducted starting from the official public communications of Romanian Ministry of Internal Affairs (MAI) regarding the fatalities declared as COVID-19 deaths by the National Institute of Public Health (INSP)^4,8^. Additionally, data from the National Center for Statistics and Informatics in Public Health were utilized to run a case-case study between the COVID-19 death population and the fatalities due to hospital pneumonia between March 22 and April 20 in the years 2016–2018 in Romania. As far as we know, this is the first study in Eastern Europe that investigates patterns of comorbidities for SARS-COV-2 fatalities.

Our principal research question is to generate hypothesis about comorbidity and burden of disease in the population deceased because of the COVID-19 virus and to investigate their association with gender and age. Additionally, the comparison between the fatalities due to COVID-19 and to pneumonia in terms of gender, age and comorbidity profiles allows us to characterize the specific medical pre-conditions of COVID-19 population.

## Results

### Population characteristics

**Figure 1:**
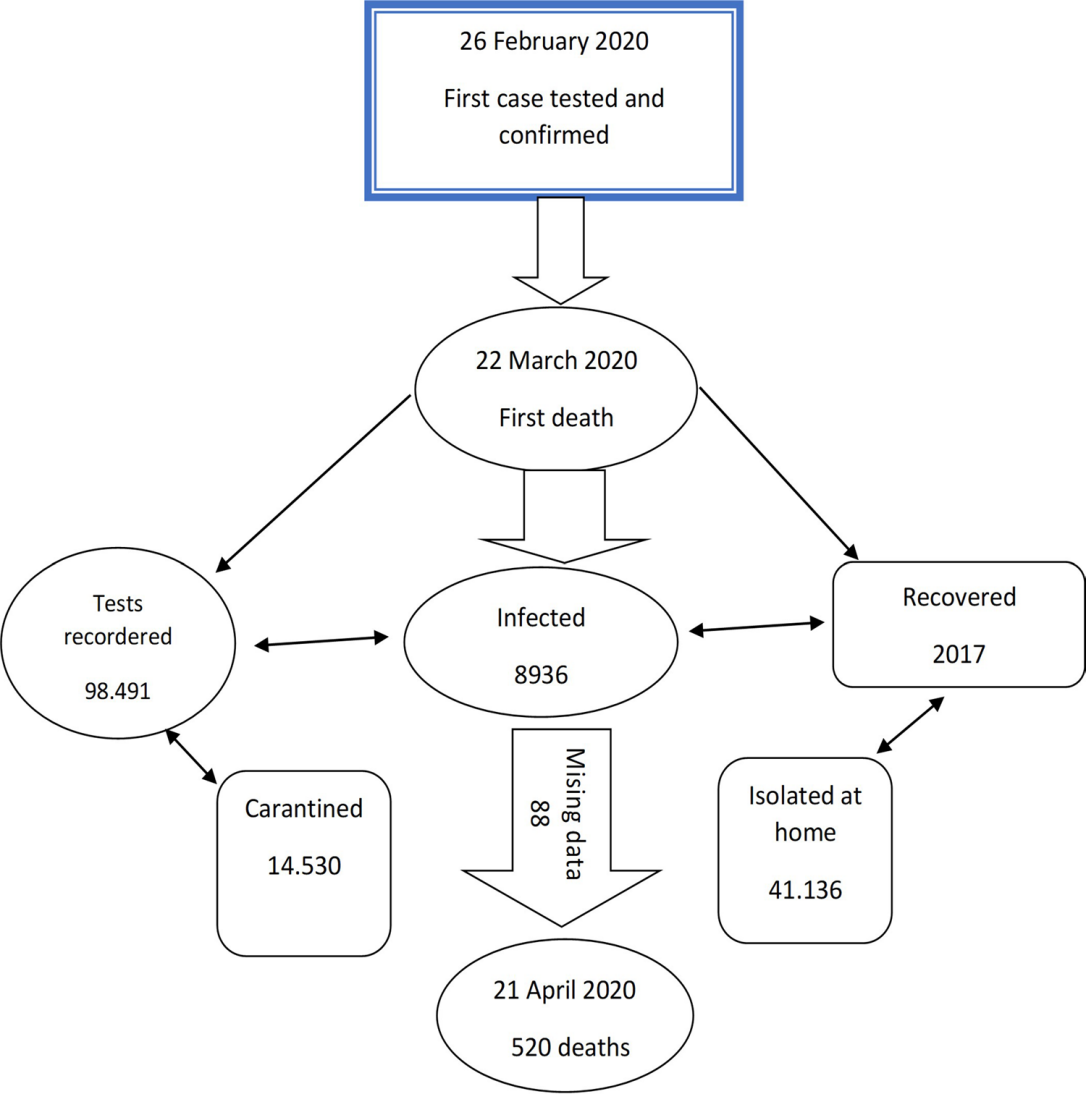
History of SARS-COV-2 pandemic in Romania up to April 20.

Figure 1 illustrates the history of the SARS-CoV-2 pandemic in Romania from February 26 until April 20, including dates, number of fatalities and number of the missing subjects due to incomplete information. The Romanian COVID-19 mortality study has a sample size of 432 patients with complete data regarding gender, age and comorbidities. It consists of a multi-case, multi-center approach focused on the mortality frequency and of a case-case evaluation with the aim of comparing hospital-pneumonia and COVID-19 mortality (see the section on the study design for the details). Our goal is to investigate the association between the mortality and comorbidities, gender, age and hospital-pneumonia based on the statistical methods presented in detail in the last section of the present article. Since the Romanian population mainly consists of 84.1% Ethnic Romanians and of a small Hungarian minority that makes up 5.4% of the population demographics that both belong to the white ethnicity, an effect of the ethnicity was not considered in this article^17^.

First, the basic descriptive statistics about our sample are presented. There are 282 (65.3%) men and 150 (34.7%) women with a statistically significant difference between the size of these two groups (Table S1). The general mean age is 67 years, with an SD of 13.1, and the median value is 68 (Table S2).

Sources of infection with deaths due to COVID-19 virus were reported in 36 counties in Romania with significant case concentration in Suceava-county n. = 67 (15.51%), Arges-county n. = 44 (10.19%) and Hunedoara-county n. = 41 (9.49%). Details regarding the spatial distribution of the distinct diseases and aggregated groups of diseases are displayed in Table S6 and Table S7 in the supplementary material.

A total of 170 different conditions and 20 groups of diseases were identified in the study, with a broader diversity of 149 conditions for men over 107 conditions for women. The diseases with the highest prevalence in the sample are hypertension, obesity, diabetes and chronic kidney disease as well as diseases of the circulatory system and nutritional or metabolic disorders.

The sample size of the pneumonia study is equal to 874 persons with complete data as to gender, age and comorbidities. The sample includes 492 (56.3%) men and 382 (43.7%) women, the average age is 73.3 years, with an SD of 13.5, and the median age is 76.

### Association between gender or age and comorbidities

One-sample proportion test indicates a statistically significant difference in the relative frequency of man and women in the sample and in the subpopulations with hypertension, diabetes, diabetes mellitus type 2, chronic kidney diseases, dependence on renal dialysis, chronic obstructive pulmonary disease, acute ischaemic heart disease or respiratory failure as displayed in Table S1. This is also valid for the subpopulations without comorbidities and for those corresponding to the aggregated group of diseases with a relative frequency of more than 5% except for diseases of the nervous system. On the other hand, the variable gender is not significantly associated with single conditions or group of illnesses except for heart failure (see Table 1).

**Table 1:** Association between gender and distinct diseases or aggregated group of diseases, as absolute numbers and as percentage.

Characteristics n,%	Men n = 282	Women n = 150	p-value
**Distinct diseases**			
Hypertension	113 40.07%	49 32.67%	0.16
Obesity	32 11.35%	21 14.00%	0.52
**Diabetes**			
Total	105 37.23%	48 32%	0.33
Diabetes mellitus, unspecified	40 14.18%	26 17%	0.47
Diabetes mellitus type 2	56 19.86%	19 13%	0.081
Diabetes mellitus type 1	9 3.19%	3 2%	0.68
Diseases of the circulatory system, unspecified	41 14.54%	27 18.00%	0.42
Supraventricular tachyarrhythmia	16 5.67%	9 6.00%	1
Heart failure, unspecified	14 4.96%	16 10.67%	**0.043**
Cerebral ischaemic stroke	16 5.67%	12 8%	0.47
Chronic kidney disease	31 10.99%	13 8.67%	0.55
Kidney failure, unspecified	17 6.03%	9 6.00%	1
Dependence on renal dialysis	29 10.28%	14 9.33%	0.88
Chronic obstructive pulmonary disease	18 6.38%	7 4.67%	0.61
Acute ischaemic heart disease, unspecified	15 5.32%	4 2.67%	0.3
Respiratory failure	15 6.32%	4 2.67%	0.3
**Aggregated group of diseases**			
No comorbidities	11 3.90%	2 1.33%	0.23
Diseases of the circulatory system	168 59.57%	88 58.67%	0.94
Endocrine, nutritional or metabolic diseases	122 43.26%	63 42.00%	0.88
Diseases of the genitourinary system	54 19.15%	25 16.67%	0.61
Diseases of the respiratory system	47 16.67%	20 13.33%	0.44
Diseases of the nervous system	35 12.41%	26 17.33%	0.21
Diseases of the digestive system	30 10.64%	14 9.33%	0.79
Neoplasms	26 9.22%	14 9.33%	1
Mental, behavioural or neurodevelopmental disorders	25 8.87%	11 7.33%	0.71
Factors influencing health status or contact with health services	37 13.12%	17 11.33%	0.7
Symptoms, signs or clinical findings, not elsewhere classified	13 4.61%	11 7.33%	0.34
Certain infectious or parasitic diseases	15 5.32%	5 3.33%	0.49

In bold the differences that reached the statistical significance.

Because age is an established risk factor for mortality, we also performed an age-stratified analysis with the lifespan split in 10-year intervals starting with age 50. The chi-squared test of association between the gender and age factors is statistically significant, with the largest male relative frequency (81.16%) between 50 and 59 years. Moreover, the factor age is also significantly associated with most of the comorbidities, with hypertension and dependence on renal dialysis following also this trend but at a significance level of 10% (see Table 2).

**Table 2:** Association between age and distinct diseases or group of diseases, as absolute numbers and as percentage.

Characteristics n, %	< 50 44 10.19%	50–59 69 15.97%	60–69 120 27.78%	70–79 128 29.93%	≥ 80 71 16.44%	p-value
**Gender**						
Male	29 66%	56 81.16%	73 60.83%	80 62.5%	44 61.97%	**0.05**
Female	15 34.09%	13 19.84%	47 39.17%	48 37.5%	27 38.03%	
**Distinct diseases**						
Hypertension	12 27.28%	21 30.43%	49 40.83%	45 35.16%	35 49.30%	0.074
Obesity	13 29.55%	9 13.04%	20 16.67%	8 6.25%	3 4.23%	**0.0001**
**Diabetes**						
Total	14 31.82%	23 33.33%	53 44.17%	48 37.5%	16 22.53%	**0.045**
Diabetes mellitus, unspecified	7 15.91%	8 11.59%	18 15.00%	24 18.75%	10 14.08%	0.74
Diabetes mellitus type 2	7 15.91%	12 17.39%	31 25.83%	20 15.63%	5 7.04%	**0.021**
Diabetes mellitus type 1	–	3 4.34%	4 3.33%	4 3.13%	1 1.40%	–
Diseases of the circulatory system, unspecified	6 13.64%	7 10.14%	18 15.00%	23 17.97%	14 19.72%	0.53
Supraventricular tachyarrhythmia	1 2.27%	–	–	12 9.38%	10 14.08%	–
Heart failure, unspecified	–	5 7.25%	10 8.33% (	7 5.47%	8 11.27%	–
Cerebral ischaemic stroke	2 4.55%	1 1.49%	9 7.50%	9 7.03%	7 9.86%	0.31
Acute ischaemic heart disease, unspecified	–	2 2.90%	7 5.83%	4 3.13%	6 8.45%	–
Chronic kidney disease	4 9.09%	3 4.35%	14 11.67%	14 10.94%	9 12.67%	0.48
Dependence on renal dialysis	3 6.82%	13 13.04%	13 10.83%	15 11.72%	3 4.23%	0.072
Kidney failure, unspecified	3 6.82%	3 4.35%	9 7.50%	8 6.25%	3 4.23%	0.87
Respiratory failure	2 4.55%	4 5.80% )	3 2.50%	4 3.13%	6 8.45%	0.33
Chronic obstructive pulmonary disease	1 2.27%	–	12 10.00%	10 7.81%	2 2.82%	–
**Aggregated group of diseases**						
Diseases of the circulatory system	14 31.82%	33 47.83%	74 61.67%	82 64.06%	53 74.65%	**0.0001**
Endocrine, nutritional or metabolic diseases	21 47.73%	30 43.48%	63 52.50%	53 41.41%	18 25.35%	**0.007**
Diseases of the genitourinary system	7 15.91%	7 10.14%	24 20.00%	29 22.66%	12 16.90%	0.27
Diseases of the respiratory system	6 13.64%	10 14.49%	18 15.00%	20 15.63%	13 18.31%	0.96
Diseases of the nervous system	3 6.82%	2 2.90%	18 15.00%	19 14.84%	19 26.76%	**0.001**
Diseases of the digestive system	4 9.09%	8 11.59%	18 15.00%	10 7.81%	4 5.63%	0.23
Neoplasms	1 2.27%	7 10.14%	12 10.00%	16 12.50%	4 5.63%	0.25
Mental, behavioural or neurodevelopmental disorders	3 6.82%	2 2.90%	6 5.00%	15 11.72%	10 14.08%	**0.049**
Factors influencing health status or contact with health services	4 9.09%	10 14.49%	15 12.50%	19 14.84%	6 8.45%	0.66
Symptoms, signs or clinical findings, not elsewhere classified	2 4.55%	3 4.35%	6 5.00%	11 8.59%	2 2.82%	0.46
Certain infectious or parasitic diseases	3 6.82%	2 2.90%	6 5.00%	4 3.13%	5 7.04%	0.63

In bold the p-values that reached the statistical significance.

### Comparison between COVID-19 and pneumonia fatalities

The comparison with pneumonia fatalities yielded that the proportion of males in the COVID-19 death sample is also significantly higher than in the pneumonia death sample as shown in Table 3. Age and the relative frequency of essential hypertension, diseases of the circulatory system, obesity, dependence of renal dialysis, and chronic kidney failure are also different between the COVID-19 and pneumonia groups. Additionally, the odd to die for COVID-19 versus hospital pneumonia is 150% greater for men than women with similar comorbidities (see Table 4). Moreover, the adjusted odds to die for COVID-19 compared to pneumonia are 22 times higher for persons with the chronic kidney failure, doubled for persons with hypertension, and almost equal for patients with type 2 diabetes.

**Table 3:** Comparison between the characteristics of the COVID-19 and pneumonia groups.

Characteristics	COVID-19	Pneumonia	p-value
**Sample size** n, %	432 100%	711 100%	
**Gender** n, %			**0.0001**
Female	150 35%	320 45%	
Male	282 65%	391 54.99%	
Age Mean (sd) Median	66.97 (13.07) 68	73.14 (13.68) 78	
**Age group** **n, %**			**0.0001**
< 50	44 10.19%	49 6.89%	
50–59	69 15.97%	51 7.17%	
60–69	120 27.78%	141 19.83%	
70–79	128 29.63%	199 27.99%	
≥ 80	71 16.44%	271 38.12%	
**Comorbidities**			
Hypertension	162 37.50%	181 25.46%	**0.0001**
Obesity	56 12.96%	63 8.86%	**0.03**
Diabetes mellitus type I	12 2.78%	5 0.70%	**0.01**
Diabetes mellitus type II	75 17.36%	145 20.39%	0.21
Dialysis	43 9.95%	4 0.56%	**0.0001**
Chronic kidney disease	43 9.95%	88 12.38%	0.21
Supraventricular tachyarrhythmia	25 5.79%	201 28.27%	**0.0001**
Congestive heart failure	12 2.78%	204 28.69%	**0.0001**
COPD	24 5.56%	72 10.13%	**0.01**
Heart failure	29 6.71%	27 3.80%	**0.03**
Kidney failure	26 6.02%	4 0.56%	**0.0001**
Respiratory failure	19 4.40%	88 12.38%	**0.0001**
Cerebral ischaemic stroke	28 6.48%	–	**0.0001**

In bold the p-values that reached the statistical significance.

**Table 4:** Logistic regression analysis for the comparison between COVID-19 and pneumonia death groups.

Covariates	Coefficient Β	OR (COVID-19 vs. pneumonia)	95% CI	p-value
Age	− 0.022	0.978	[0.968–0.988]	**0.0001**
Gender (0 = female)	0.414	1.513	[1.145–2.000]	**0.01**
Hypertension	0.740	2.096	[1.560–2.818]	**0.0001**
Obesity	0.266	1.305	[0.843–2.019]	0.23
Diabetes type 2	− 0.355	0.701	[0.494–0.996]	**0.049**
Dialysis	3.104	22.290	[7.244–68.590]	**0.0001**
Chronic kidney disease	− 0.522	0.593	[0.369–0.955]	**0.03**
Heart failure	1.095	2.990	[1.612–5.546]	**0.0001**
COPD	− 0.730	0.482	[0.284–0.817]	**0.01**
Supraventricular tachyarrhythmia	− 1.687	0.185	[0.116–0.295]	**0.0001**
Respiratory failure	− 1.045	0.352	[0.203–0.609]	**0.0001**
Intercept	0.884	2.420		**0.02**

In bold the p-values that reached the statistical significance.

### The burden of disease and survival probability

We also extended the analysis to the coexistence of multiple health conditions, and we focused on the multimorbidity. This has a mean value of 2.109, an SD of 1.66 and a median value of 2 in our study. The multimorbidity factor with levels 0, 1, 2, 3, 4 or more than 5 for single or aggregated comorbidities is significantly associated with the factor age but not with gender, confirming the pattern displayed at the level of single conditions and group of diseases (Tables S3 and S4).

The Charlson comorbidity index (CCI) had a mean of 1.324, SD of 0.95 and median of one indicating that the severity of the underlying medical condition in the study was mainly mild. This was confirmed by the high relative frequency of 91.20% for the mild level for the factor CCI. There were no significant differences between males and females or among age groups with respect to CCI (Tables S3 and S4). Moreover, 1-year survival probability averaged out at 81%, and 50% of the study individuals had a prognosis for it of 85%.

### Co-occurrence patterns

The co-occurrence of the diseases is of great importance since the mean multimorbidity of 2.109 indicates that not single but pair of diseases are pre-conditions in our mortality study. Pearson's phi coefficient is a robust measure of the comorbidity association, and its values for diseases in general and separated for men and women are displayed in the Table S5. Different co-occurrence patterns emerge between men and women with the striking lack of the correlation between pneumonia or respiratory failure, respectively stroke and dissociative neurological symptom disorder in the female profile. The largest value of the parameter phi of 0.47 respectively 0.40 for female and 0.59 for male corresponds to kidney failure and dependence to dialysis, while the latter is also correlated with chronic kidney disease as the correlation lies by 0.30 in general and 0.38 in men. They are followed by pneumonia or respiratory failure with phi equal to 0.45 in general and to 0.55 in male and by stroke and dissociative neurological symptom disorder with a correlation coefficient of 0.41 in the whole study and of 0.49 in men. Diabetes mellitus type 2 is correlated with hypertension not only for the whole study (phi = 0.11) but also in the men (phi = 0.10) and women subsamples (phi = 0.11). Hypertension appears mainly together with stroke with phi equal to 0.15, 0.16 respectively 0.14 for the whole study, men and women.

**Figure 2:**
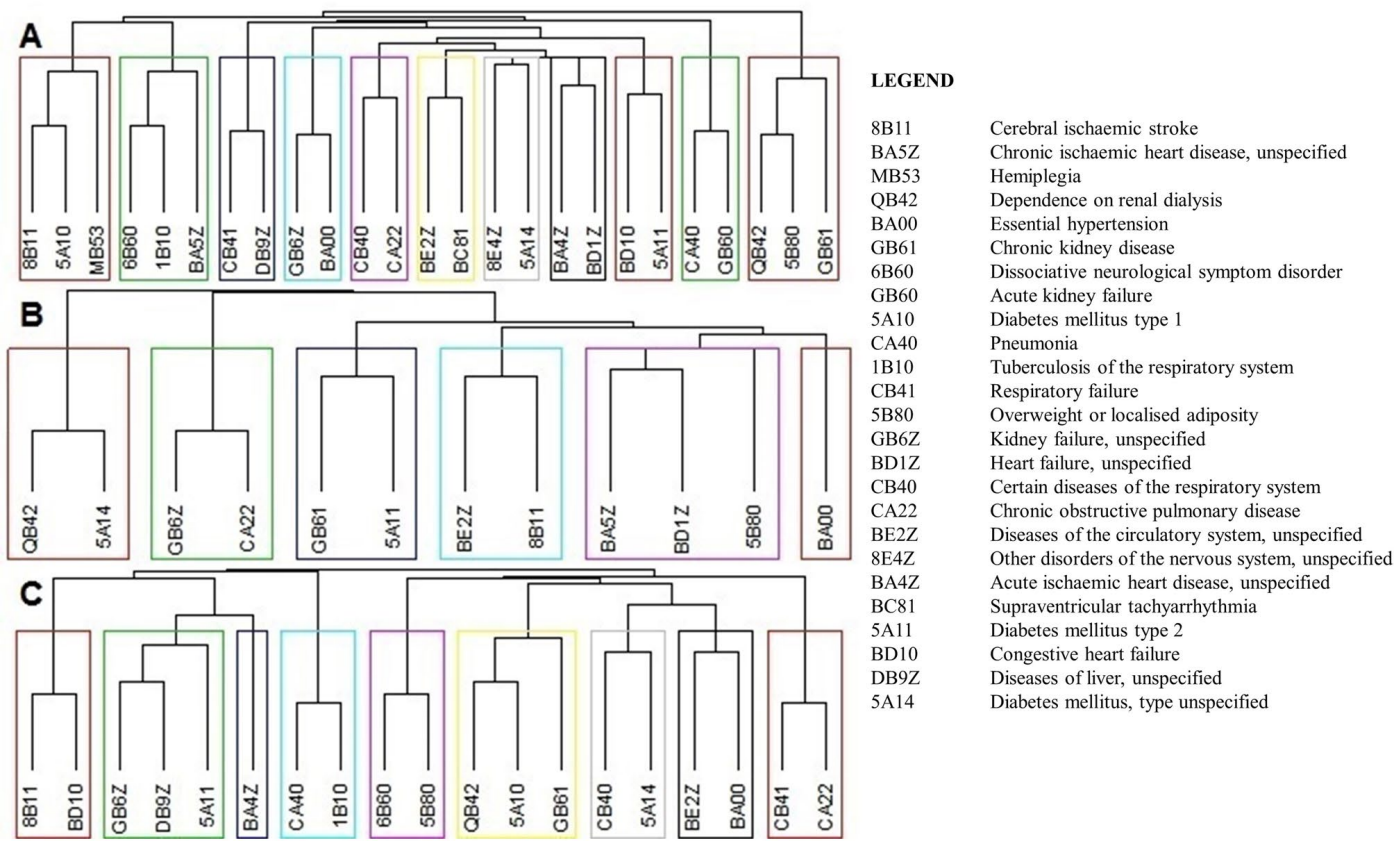
Co-occurrence clusters for All Subjects (**A**), Women (**B**) and Men (**C**).

Finally, a comorbidity network analysis rounded off our exploratory investigation. This identified 11 clusters in the general study, 9 clusters in male, and 6 clusters in female subgroups, each including two or three diseases reinforcing the previous results about pair of diseases as pre-condition for COVID-19 fatalities (see Fig. 2).

## Discussion

The high relative frequency of men in the whole study (65.3% males vs. 34.7% females, p-value < 0.001) and in all subpopulations with distinct diseases and aggregated group of diseases shows that an important risk factor for death by COVID-19 in Romania is being male. This is consistent with other studies about predictors for severity and mortality due to infection with SARS-CoV 2 in other populations^10,18^.

In addition, the comorbidities with the highest prevalence in our study, such as hypertension (37.5%), diabetes (35.4%), obesity (12.27%) and chronic kidney disease (10.19%) as well as diseases of the circulatory system (59.26%) and nutritional or metabolic disorders (42.82%), had similar frequencies with those reported in Chinese or American studies but contradicts the findings of the Italian CORIST study^10,19–21^. Indeed, the percentages that we found for the distinct comorbidities are consistent to those reported for China; for instance. Li et al., found that the majority of patients deceased in the hospital (N = 128) were men (73%), and that the most prevalent comorbidities associated with COVID-19 mortality were hypertension (47%) and diabetes (13%), while Zhou et al. reported that, among 54 fatalities for COVID-19, 62% were men, and the most prevalent comorbidities were hypertension (30%), diabetes (19%) and coronary heart disease (8%)^22,23^. These diseases also display significant differences in the relative frequency of men vs. women in our study in Romanian population: 74.67% men vs. 25.33% women for diabetes mellitus type 2, 69.75% men vs. 30.25% women for hypertension, 70.45% men vs. 29.55% women for chronic kidney disease, as well as 65.62% vs. 34.38% respectively 65.95% vs. 34.95% for diseases of the circulatory system and for nutritional or metabolic disorders. Almost all the comorbidities in our study are not associated with gender but with age, as also previously reported in other populations^24,25^.

We found in the present study the highest number of deaths in the age group between 70–79 years (N = 128, 29.63%), followed by the age group 60–69 years (N = 120, 27.78%) and the majority were men (62.5% men vs. 37.5% women). The Romanian age-specific mortality statistics are similar to the estimates of crude-case fatality from Italy, Spain or Germany at the beginning of April, and in contrast to the United Kingdom, where the death count was higher in 65+ years population cluster with a relative frequency of 44% for persons aged 65–79 years and 46% for the 80+ age group^4–8,26^. The Romanian age structure of the COVID-10 fatalities is similar to the Chinese and lower than the Italian group^9,25^. Subjects younger than 50 years are less susceptible to die by COVID-19 with a relative frequency of 10% as reported in other articles, and those over 70 years are almost half of the study participants (46%)^27^. In the survey by Du et al. it was found that subjects who died for SARS-CoV-2 infection were older than 65 years, and that the main comorbidities related to death were hypertension and CVD^28^. On the other side, in ten European countries and Canada, fatalities due to COVID-19 for individuals younger than 65 years represented 4.8–9.3% of all. Only 13.0% of the COVID-19 fatalities in the UK and 7.8–23.9% in the USA were younger than 65 years. On the other hand, the prevalence for subjects older than 80 years was 54–69% in Europe, 67% in Canada and 36–63% in US^27^. Another important meta-analysis made by Verity et al. has shown that 13.4% persons older than 80 with more than one comorbidity died for SARS-CoV-2 and that the estimated case fatality rate was 1.38% for 38 countries^29^. Also, in the Spanish study by Perez-Tanoira et al., men older than 85 years represented 27% of their cohort (N = 108) and the most prevalent comorbidity was coronary heart disease, with a crude mortality rate of 19%^30^.

Statistics displayed in Table S1 and Table 3 show that gender and age are decisive factors for COVID-19 mortality in our Romanian population. This is of great importance for tailored personalized medicine, where treatment management needs to be adapted to patients’ characteristics, and in the epidemiology, where population stratification is the key factor for the identification of the highest risk subgroups.

In the whole world, the incidence of COVID-19 and its mortality rate is higher for people with diabetes and/or obesity. The precise mechanisms of SARS-CoV-2 infection are still not fully elucidated, but it has been suggested that this may be due to the chronic inflammatory state and/or insulin-resistance^31,32^. In our study, diabetes mellitus was classified as type-1, type-2 and unspecified. The unspecified diabetes is most probably the form of type-2 diabetes, since most of the deceased people are between 50 and 79 years and the incidence of the diabetes of type 2 in Romania is extremely higher than type-1, as also shown in the Predatorr study^15^. Hence, we can assume that the relative frequency of diabetes type-2 was higher than hypertension for the three clusters of age 50–59, 60–69 and 70–79 years.

The association between age and different comorbidities suggest the existence of age-stratified positive and negative markers for COVID-19 mortality. Generally, obesity correlates positively with diabetes^30,31^, although this is confirmed in our study only in the group of youngest age. with a prevalence of 29.55%. Hence, we can interpret obesity as a negative marker for the severity of COVID-19 infection in combination with age below 50 years. In addition, we found the following negative markers: for the age group 50–59 years diabetes (33.33%), for the age group 60–69 years diabetes (44.17%) and hypertension (35.16%), for the age group 70–79 diabetes (44.17%), hypertension (35.16%) and chronic kidney diseases (17.87%), and for the age group over 80 years hypertension (49%), diabetes (22.53%) and the diseases of the circulatory system (19.72%). Further, the main risk factors for death regarding aggregated groups of diseases were the following: the endocrine diseases (diabetes, obesity) with a prevalence of 47.73% for subjects under 50 years, and diseases of the circulatory system, with a prevalence between 47.83 and 74.65% for subjects in all the other age groups.

The prevalence of respiratory failure and chronic pulmonary diseases in our study was almost the lowest compared to the rest of the comorbidities but it was similar with the data reported elsewhere^22,23^. Diseases of the respiratory system were more frequent in the age group over 80 years (18.31%) and had higher prevalence in men (16.67%) versus women (13.33%). This is somewhat surprising since pneumonia had a higher prevalence in the Romanian population in the last three years. Usually, patients with comorbidities from this group of diseases are more vulnerable to viral respiratory infections^33^. Moreover, it is known that a symptom for the infection with SARS-CoV 2 is an atypical pneumonia with a variable degree of severity^34^. Since pneumonia could be a negative prognostic factor for COVID-19 patients^35^, we investigated the relation between the comorbidities of the two diseases. The results show significant differences between these two diseases as shown in Table 4. The hypothesis about the diseases of the respiratory system and the neoplasms as prognosis factors for the mortality through COVID-19 is not confirmed in our study. Therefore, it is likely that patients died because of the impact and virulence of the SARS-CoV-2 infection over comorbidities like hypertension, diabetes mellitus or obesity, in combination with age and gender^36^.

Surprisingly, the average multimorbidity for the COVID-19 fatalities is low but confirms previous findings about infected populations or groups with a severe form of the disease^19^.

The high relative frequency of the mild CCI corroborates this conclusion regarding subjects with a low burden of disease. Our prognosis regarding the survival probability confirms other observations about the estimated year's life lost by the COVID-19 fatalities being "considerably more than the "1–2 years" as presented in^37^.

This is the first article where the co-occurrence profiles are investigated with the help of Pearson's co-occurrence coefficient and where co-occurrence patterns are explicitly described through clustering. Our findings on clustering data are not surprising since diabetes appears mainly with cardiovascular diseases, and kidney diseases with dialysis. Also, the sparsity of respiratory diseases, particularly in the women subgroup, should be further investigated.

In conclusion, our study characterizes comorbidities of COVID-19 fatalities in Romania and their association with gender and age. Most of our results are consistent with previous studies from other populations, and the main differences in our Romanian population lie in the lower age of the deceased patients, on their small multimorbidity count and their high 1-year survival probability. In addition, the comparison with the pneumonia group offers a new perspective on the medical pre-conditions of SARS-CoV 2.

Fortunately, we registered a low incidence of COVID-19 deaths in Romania until 20th of April 2020, and future studies are needed to determine precisely the pathogenic mechanism that affected these patients, especially males with hypertension, diabetes, chronic kidney disease or obesity and potential protective conditions like those suggested in^38–41^.

## Methods

### Study design

#### The cohort

The Romanian COVID-19 mortality sub-study was conducted between March 22 and April 20, 2020, with the aim to explore the comorbidity patterns in the patients who died because of this new virus. A multiple-case, multiple-center design was chosen to answer this research question with data collected from the official public notifications of MAI concerning the COVID-19 deaths registered by the INSP^3,4,8^.

Initially, 451 COVID-19 deaths were reported by INSP from the beginning of the epidemic until April 20 (Fig. 1).

The list was completed afterwards with 69 additional cases due to the delay in the reporting process from some centers of COVID-19 infection. The official report included information about gender, age, county of residence, history of hospitalization and diagnostics, and/ or comorbidities. Only cases with information about gender, age and comorbidities were included in the study due to the main focus of the research^42^. Eventually, 432 patients who fulfilled these criteria were kept in the Romanian COVID-19 mortality sub-study. Since we aim at an analytic and not statistical generalization of the results regarding the comorbidity patterns, statistical testing was just serving exploratory purposes^42^. Hence, the complete-case analysis is justified in this case both by purpose and by the state of things.

### Case-case study

Additionally, we run a case-case study utilizing data regarding the deaths due to hospital pneumonia between March 22 and April 20 in the years 2016–2018 extracted from the administrative database of the INSP.

The study was carried out with the approval of the Local Bioethics Council of the INSP (number 6342/04 MAY 2020) and with the permission of the Local Bioethics Council of the “Carol Davila” University of Medicine, Bucharest Romania (9984/11 MAY 2020).

### Statistical analysis

An exploratory data analysis was applied to identify general, spatial-, gender—or age-specific comorbidity patterns in our study. In a first step, the various conditions were sorted according to the standard diagnosis tool ICD-11 (The International Classification of the Diseases) as it was recommended for the mortality and morbidity statistics by the WHO^43^. The ICD-11 taxonomy includes categories of single diseases or the aggregated groups of diseases. We investigated the comorbidities at both levels of aggregation because the study included a large number of distinct conditions with low prevalence. The statistical analysis was done with the help of the statistical software R version 3.6.2.

Continuous variables were expressed as mean, standard deviation (SD) and median. We focused in this paper on comparing the individual crude relative frequencies of the single and aggregated comorbidities between different gender and age groups. Since age is a significant risk factor for mortality, we investigated the frequency of the comorbidities stratified on ten-year intervals starting with the 50+ age groups. Categorical variables were tabulated in frequency (n) and relative frequency (%). A binomial and a chi-squared test for one proportion (only the last was reported) and a chi-squared test for equal proportions were applied when it was appropriate at the 5% level of significance.

The post-hoc tests were not corrected for multiple comparisons since the focus of our study was on generating hypotheses for single diseases or group of diseases and not on an experiment-wise approach. The spatial distribution of the COVID-19 mortality corresponded to the country-specific administrative units– counties and was illustrated by single or aggregated disease frequency and relative frequency (%).

The association between different comorbidities and the odds ratio between COVID-19 and pneumonia deaths was investigated with the help of the logistic regression, and the results were reported as coefficients β, as point estimates for odds ratio and their 95% confidence intervals.

Multimorbidity was computed for each person in the sample since this is a good indicator of the burden of disease^44,45^ and transformed in a multimorbidity factor with levels 0, 1, 2, 3, 4, or more the 5 for single or aggregated comorbidities.

A controversial issue regarding the epidemic of COVID-19 is the burden of disease in the deceased population and the hypothetical survival probability in the case that these people would not have been infected with this virus. A simplified version of the Charlson Comorbidity Index (CCI) was calculated on a scale from 0 to 8, as indicated in^24^ for an assessment of the individual disease severity. We introduced the CCI factor with two levels (mild for CCI < 3 and severe for CCI ≥ 3), and we tested its differences between gender and age groups by a chi-squared test.

Following the same paper^24^, we adapted their statistical model for the individual prognosis of the death risk based on the reported hazard ratio for the age levels and for the CCI in multiple European studies as explained in the section 8 of the supplementary material. This gave us a prognosis for the mean, the standard deviation and the median of the crude 1-year death risk and survival probability.

The next step in the analysis of the COVID-19 comorbidities concerns the co-occurrence of the diseases. This was explored with the help of the Pearson's phi coefficient as defined and investigated, e.g. in^46^ and computed for each pair of single or aggregated diseases with an incidence of at least 4. A value of 1 means a "perfect match", 0 means no co-occurrence and negative values up to − 1 means "inverse occurrence".

A comorbidity network approach was considered to investigate the relationships between diseases. We computed the dissimilarity between the conditions studied in the previous step as one minus the Pearson's phi. Hence, a value of 1 corresponds to "no association" and values larger than 1 to "inverse association". The hierarchical classification was performed through an agglomerative clustering algorithm with the Ward method since this proved to have the largest agglomeration coefficient. Furthermore, the dendrogram was cut at nodes corresponding to branch length of 1 based both on a visual inspection and on predefined length of the branch.

This study has several limitations. We considered only the deaths up to April 20 when the peak of the first pandemic wave was reached in Romania. Moreover, total case design was used because of the missing data in official public communications.

### Disclaimer

This research did not receive any specific grant from funding agencies in the public, commercial, or not-for-profit sectors.

### Ethical compliance

This research was carried out in accordance with the relevant guidelines and regulations.

### Informed consent

We have obtained informed consent from all participants and/or their legal guardians.

## Supplementary Information

Supplementary Information.
