# Supplementary material for: Death by SARS-CoV 2: a Romanian COVID-19 multi-centre comorbidity study
Source: Sci Rep. 2020 Dec 10;10:21613. doi: 10.1038/s41598-020-78575-w (PMC7730445; doi:10.1038/s41598-020-78575-w)
Supplement: Supplementary file 1 — Supplementary Information. [file 41598_2020_78575_MOESM1_ESM.pdf]

Death by SARS-CoV 2 - a Romanian COVID-19 multi-centre comorbidity study

Authors: Anca Pantea Stoian[1,2]*MD,PhD, Mihaela Pricop-Jeckstadt[3,4]*PhD, Adrian Pana[5]*, Bogdan-Vasile Ileanu[5], Ruxandra Schitea[6]*, Marius Geanta[6] MD,PhD, Doina Catrinoiu[7]MD,PhD, Andra Iulia Suceveanu[7]MD,PhD, Cristian Serafinceanu[1,2]MD,PhD, Silviu Pituru[1]MD,PhD, Catalina Poiana[1]MD,PhD, Bogdan Timar[8]MD,PhD, Cornelia Nitipir[1]MD,PhD, Simona Parvu[1]MD, Andreea Arsene[1]MD,PhD, Laura Mazilu[7]MD,PhD, Antonela Toma[4,9]PhD, Razvan Hainarosie[1,10]MD,PhD, Antonio Ceriello[11]*MD,PhD, Manfredi Rizzo[12,13]*MD,PhD, Viorel Jinga[1]MD,PhD.

[1]University of Medicine and Pharmacy "Carol Davila", Bucharest, Romania, 37 Dionisie Lupu Str., 020021, Bucharest, Romania;

[2]National Institute of Diabetes, Nutrition and Metabolic Diseases "N. C. Paulescu", 5-7. Ion Movila Str,030167, Bucharest, Romania;

[3] Department of Applied Mathematics, University Politehnica of Bucharest, Splaiul Independentei 313, 060042, Bucharest, Romania;

[4]Center for Research and Training in Innovative Techniques of Applied Mathematics in Engineering - ~*Traian Lalescu*~ -(CiTi), University Politehnica of Bucharest, Splaiul Independentei 313, 060042, Bucharest, Romania;

[5]Center for Health Outcomes & Evaluation, Splaiul Unirii 45, 030126, Bucharest, Romania;

[6]Center for Innovation in Medicine, Bd. Theodor Pallady, no. 42J, room 1719, 032266, Bucharest, Romania;

[7]Department of Internal Medicine, Faculty of Medicine, Universitatea Ovidius University C2 Street, 900527, Constanta, Romania;

[8] "Victor Babes" University of Medicine and Pharmacy, 2 Eftimie Murgu, 300041, Timisoara, Romania;

[9]Department of Mathematical Methods and Models, University Politehnica of Bucharest, Splaiul Independentei 313, 060042, Bucharest, Romania;

[10]Prof. Dr D. Hociota" Institute of Phonoaudiology and Functional ENT Surgery, 21st Mihail Cioranu Street, 061344, Bucharest, Romania;

[11]IRCCS MultiMedica, 300 Milanese str, 20099 Sesto San Giovanni MI Milan, Italy;

[12]Division of Endocrinology, Diabetes and Metabolism, Department of Medicine, University of South Carolina, 6311 Garners Ferry Rd, SC 29209 Columbia, SC, USA;

[13]Department of Health Promotion, Mother and Child Care, Internal Medicine and Medical Specialties, University of Palermo, 61 Piazza Marina str, 90133, Palermo, Italy.

*These authors contributed equally to the present work.

Correspondent author: Mihaela Pricop-Jeckstadt, Department of Applied Mathematics, University Politehnica of Bucharest, Splaiul Independentei 313, 060042, Center for Research and Training in Innovative Techniques of Applied Mathematics in Engineering (CiTi), University Politehnica of Bucharest, Splaiul Independentei 313, 060042, Bucharest, Romania.Email:Mihaela.Pricop@upb.ro Telephone: +04021402-9353

Supplementary Appendix

Table of Contents

1. Supplementary Table S1..........................pg.2

2. Supplementary Table S2..........................pg.3

3. Supplementary Table S3................................................pg.6

4. Supplementary Table S4.......................pg.8

5. Supplementary Table S5........................pg.10

6. Supplementary Table S6 (with legend)........................pg.15

7. Supplementary Table S7 (with legend)........................pg.18

8. Section 8: Death risk and survival probability........................pg.22

1. Supplementary Table S1. Prevalence of distinct diseases and aggregated group of diseases, as counts and as percentage. Frequency of the male and female groups in each subpopulation with distinct diseases or aggregated group of diseases (by row, as counts and as percentage). In bold the p-values for the differences in the relative frequency per disease or per aggregated group of diseases between male and female that reached the statistical significance with respect to the one-sample proportion test.

| Characteristics<br>n,% | | Total<br>n, % | Male | Female | p-value |
|---|---|---|---|---|---|
| Sample size | | 432<br>100% | 282<br>65.3% | 150<br>34.7% | 0.0001 |
| Diseases | | | | | |
| Hypertension | | 162<br>37.50% | 113<br>69.75% | 49<br>30.25% | 0.0001 |
| Obesity | | 53<br>12.27% | 32<br>60.38% | 21<br>39.62% | 0.17 |
| Diabetes | Total | 153<br>35.42% | 105<br>68.63% | 48<br>31.37% | 0.0001 |
| | Diabetes mellitus, unspecified | 66<br>15.28% | 40<br>60.61% | 26<br>39.39% | 0.11 |
| | Diabetes mellitus type 2 | 75<br>17.36% | 56<br>74.67% | 19<br>25.33% | 0.0001 |
| | Diabetes mellitus type 1 | 12<br>2.78% | 9<br>75% | 3<br>25% | 0.15 |
| Diseases of the circulatory system, unspecified | | 68<br>15.74% | 41<br>60.29% | 27<br>39.71% | 0.11 |
| Supraventricular tachyarrhythmia | | 25<br>5.79% | 16<br>64% | 9<br>36% | 0.23 |
| Heart failure, unspecified | | 30<br>6.94% | 14<br>46.67% | 16<br>53.33% | 0.86 |
| Cerebral ischaemic stroke | | 28<br>6.48% | 16<br>57.14% | 12<br>42.86% | 0.57 |
| Chronic kidney disease | | 44<br>10.19% | 31<br>70.45% | 13<br>29.55% | *0.01* |
| Kidney failure, unspecified | | 26<br>6.02% | 17<br>65.38% | 9<br>34.62% | 0.17 |
| Dependence on renal dialysis | | 43<br>9.95% | 29<br>67.44% | 14<br>32.56% | *0.033* |
| Chronic obstructive pulmonary disease | | 25<br>5.79% | 18<br>72% | 7<br>28% | *0.0462* |
| Acute ischaemic heart disease, unspecified | | 19<br>4.40% | 15<br>78.95% | 4<br>21.05% | *0.022* |
| Respiratory failure | | 19<br>4.40% | 15<br>78.95% | 4<br>21.05% | *0.02* |
| Aggregated group of diseases | | | | | |
| No comorbidities | | 13<br>3.01% | 11<br>84.62% | 2<br>15.38% | 0.027 |
| Diseases of the circulatory system | | 256<br>59.26% | 168<br>65.62% | 88<br>34.38% | 0.0001 |

| | | | | |
|---|---|---|---|---|
| Endocrine, nutritional or metabolic diseases | 185<br>42.82% | 122<br>65.95% | 63<br>34.05% | 0.0001 |
| Diseases of the genitourinary system | 79<br>18.29% | 54<br>68.35% | 25<br>31.65% | 0.002 |
| Diseases of the respiratory system | 67<br>15.51% | 47<br>70.15% | 20<br>29.85% | 0.002 |
| Diseases of the nervous system | 61<br>14.12% | 35<br>57.38% | 26<br>42.62% | 0.31 |
| Diseases of the digestive system | 44<br>10.19% | 30<br>68.18% | 14<br>31.982% | 0.024 |
| Neoplasms | 40<br>9.26% | 26<br>65% | 14<br>35% | 0.082 |
| Mental, behavioural or neurodevelopmental disorders | 36<br>8.33% | 25<br>69.44% | 11<br>30.56% | 0.03 |
| Factors influencing health status or contact with health services | 54<br>12.50% | 37<br>68.52% | 17<br>31.48% | 0.010 |
| Symptoms, signs or clinical findings, not elsewhere classified | 24<br>5.60% | 13<br>54.17% | 11<br>45.83% | 0.84 |
| Certain infectious or parasitic diseases | 20<br>4.62% | 15<br>75% | 5<br>25% | 0.044 |

2. Supplementary Table S2.: Study participants and severity of disease (total, male, female). In bold the differences in the relative frequency (by row) between male and female that reached the statistical significance with respect to the one-sample proportion test.

| Characteristic | Total | Male | Female | p-value |
|---|---|---|---|---|
| Age in years<br>mean (sd)<br>median | <br>67 (13.1)<br>68 | <br>66.4 (13)<br>67 | <br>68.1 (13.1)<br>69.5 | |
| Disease diversity<br>n, % | 170<br>100% | 139<br>82% | 107<br>63% | |
| Multimorbidity<br>mean (s.d.)<br>median | 2.583 (1.58)<br>2 | 2.58 (1.61)<br>2 | 2.59 (1.52)<br>2 | |
| Multimorbidity Factor<br>n, % | | | | |

| | | | | |
|---|---|---|---|---|
| With 0 comm | 13<br>3% | 11<br>85% | 2<br>15% | *0.027* |
| With 1 comm | 112<br>26% | 71<br>63% | 41<br>37% | *0.006* |
| With 2 comm | 104<br>24% | 67<br>64% | 37<br>36% | *0.005* |
| With 3 comm | 100<br>23% | 65<br>65% | 35<br>35% | *0.004* |
| With 4 comm | 61<br>14% | 41<br>67% | 20<br>33% | *0.01* |
| More than 5 comm | 42<br>10% | 27<br>64% | 15<br>36% | 0.09 |
| Aggregated group of diseases | | | | |
| Multimorbidity<br>mean (s.d.)<br>median | 2.109 (1.18)<br>2 | 2.14 (1.21)<br>2 | 2.05 (1.14)<br>2 | |
| Multimorbidity factor<br>n, %, | | | | |
| With 0 comm | 13<br>3% | 11<br>85% | 2<br>15% | *0.027* |
| With 1 comm | 140<br>32% | 88<br>63% | 52<br>37% | *0.003* |
| With 2 comm | 139<br>32% | 83<br>60% | 56<br>40% | *0.027* |
| With 3 comm | 88<br>21% | 64<br>73% | 24<br>27% | 0.0001 |
| With 4 comm | 38<br>9% | 27<br>71% | 11<br>29% | *0.015* |
| More than 5 comm | 14<br>3% | 9<br>64% | 5<br>36% | 0.42 |

| | | | | |
|---|---|---|---|---|
| | | | | |
| CCI<br>mean (s.d.)<br>median | 1.324 (0.95)<br>1 | 1.35 (0.98)<br>1 | 1.27 (0.89)<br>1 | |
| | | | | |
| CCI factor<br>n, % | | | | |
| mild | 394<br>91.20% | 255<br>65% | 139<br>35% | 0.0001 |
| severe | 38<br>8.80% | 27<br>71% | 11<br>29% | *0.015* |
| Prognosis | | | | |
| 1-year mortality<br>mean (sd)<br>median | 0.19<br>(0.03)<br>0.15 | 0.24<br>(0.03)<br>0.19 | 0.10<br>(0.01)<br>0.08 | |
| 1-year survival probability<br>mean (sd)<br>median | 0.81<br>(0.03)<br>0.85 | 0.76<br>(0.03)<br>0.81 | 0.90<br>(0.01)<br>0.92 | |

3. Supplementary Table S3. Association between gender and severity of diseases, as absolute numbers and as percentage (by column). In bold the differences in percentage that reached the statistical significance with respect to the chi-squared test.

| Gender, n | Male<br>n=282 | Female<br>n=150 | |
|---|---|---|---|
| Characteristic | | | p-value |
| Diseases | | | |
| Multimorbidity Factor<br>n, %, | | | 0.78 |
| With 0 comm | 11<br>3.90% | 2<br>1.33% | 0.23 |
| With 1 comm | 71<br>25.18% | 41<br>27.33% | 0.71 |
| With 2 comm | 67<br>23.76% | 37<br>25.67% | 0.93 |
| With 3 comm | 65<br>23.05% | 35<br>23.33% | 1 |
| With 4 comm | 41<br>14.54% | 20<br>13.33% | 0.84 |
| More than 5 comm | 27<br>9.57% | 15<br>10% | 1 |
| Aggregated group of diseases | | | |
| Multimorbidity<br>mean (s.d.)<br>median | 2.14 (1.21)<br>2 | 2.05 (1.14)<br>2 | |
| Multimorbidity factor<br>n, %, | | | 0.21 |
| With 0 comm | 11 | 2 | 0.23 |

| | | | |
|---|---|---|---|
| | 3.90% | 1.33% | |
| With 1 comm | 88<br>31.21% | 52<br>34.67% | 0.53 |
| With 2 comm | 83<br>29.43% | 56<br>37.33% | 0.12 |
| With 3 comm | 64<br>22.70% | 24<br>16.00% | 0.13 |
| With 4 comm | 27<br>9.57% | 11<br>7.33% | 0.55 |
| More than 5 comm | 9<br>3.19% | 5<br>3.33% | 1 |
| CCI<br>mean (s.d.)<br>median - IQR | 1.35 (0.98)<br>1 - 1 | 1.27 (0.89)<br>1 - 1 | |
| | | | |
| CCI factor<br>n, % | | | 0.55 |
| mild | 255<br>90.43% | 139<br>92.67% | 0.55 |
| severe | 27<br>9.57% | 11<br>7.33% | 0.55 |
| Prognosis | | | |
| 1-year mortality<br>mean (sd)<br>median | 0.24<br>(0.03)<br>0.19 | 0.10<br>(0.01)<br>0.08 | |
| 1-year survival probability<br>mean (sd)<br>median | 0.76<br>(0.03)<br>0.81 | 0.90<br>(0.01)<br>0.92 | |

4. Supplementary Table S4: Association between the factor age and the severity of disease, as absolute numbers and as percentage (by column). In bold the differences in percentage that reached the statistical significance with respect to the chi-squared test

| Age (stratified)<br>n | <50<br>44 | 50-59<br>69 | 60-69<br>120 | 70-79<br>128 | >=80<br>71 | p-value |
|---|---|---|---|---|---|---|
| Characteristic | | | | | | |
| Diseases | | | | | | |
| Disease diversity<br>n, % | 42<br>24.71% | 61<br>35.88% | 100<br>58.83% | 103<br>60.59% | 54<br>31.76% | |
| Multimorbidity<br>mean (s.d.)<br>median | 2.07 (1.66)<br>2 | 2.17 (1.45)<br>2 | 2.82 (1.63)<br>3 | 2.68 (1.50)<br>3 | 2.73 (1.60)<br>2 | |
| Multimorbidity factor<br>n ,% | | | | | | *0.003* |
| With 0 comm | 4<br>9% | 4<br>6% | 2<br>2% | 1<br>1% | 2<br>3% | *0.035* |
| With 1 comm | 17<br>39% | 24<br>35% | 2<br>2% | 27<br>21% | 15<br>21% | 0.058 |
| With 2 comm | 11<br>25% | 15<br>22% | 26<br>22% | 32<br>25% | 20<br>28% | 0.86 |
| With 3 comm | 3<br>7% | 16<br>23% | 26<br>22% | 43<br>34% | 12<br>17% | 0.003 |
| With 4 comm | 6<br>14% | 6<br>9% | 18<br>15% | 16<br>13% | 15<br>21% | 0.30 |
| More than 5 comm | 3<br>7% | 4<br>6% | 19<br>16% | 9<br>7% | 7<br>10% | 0.099 |
| Aggregated Comorbidities | | | | | | |
| Multimorbidity | 1.70 (1.29) | 1.77 (1.02) | 2.29 (1.29) | 2.26 (1.10) | 2.11 (1.13) | |

| | | | | | | |
|---|---|---|---|---|---|---|
| mean (s.d.)<br>median | 1 | 2 | 2 | 2 | 2 | |
| Multimorbidity factor<br>n ,% | | | | | | *0.014* |
| With 0 comm | 4<br>9% | 4<br>6% | 2<br>2% | 1<br>1% | 2<br>3% | *0.035* |
| With 1 comm | 21<br>48% | 27<br>39% | 37<br>31% | 35<br>27% | 20<br>28% | 0.079 |
| With 2 comm | 11<br>25% | 24<br>35% | 35<br>29% | 41<br>32 % | 28<br>39% | 0.48 |
| With 3 comm | 2<br>5% | 10<br>15% | 25<br>21% | 38<br>30% | 13<br>18% | *0.004* |
| With 4 comm | 5<br>11% | 3<br>4% | 15<br>13% | 9<br>7% | 6<br>8% | 0.33 |
| With more than 5 comm | 1<br>2% | 1<br>1% | 6<br>5% | 4<br>3% | 2<br>3% | 0.72 |
| CCI | | | | | | |
| Charlson comorbidity index (CCI)<br>mean (s.d.)<br>median | 0.84 (0.94)<br>1 | 1.06 (0.78)<br>1 | 1.48 (1.05)<br>1 | 1.51 (0.89)<br>1 | 1.30 (0.87)<br>1 | |
| CCI factor<br>n ,% | | | | | | 0.007 |
| Mild comorbidities | 42<br>95.45% | 69<br>100% | 103<br>85.83% | 113<br>88.28% | 67<br>94.37% | 0.007 |
| Severe comorbidities | 2<br>4.55% | - | 17<br>14.17% | 15<br>11.72% | 4<br>5.63% | 0.007 |
| 1-year prognosis | | | | | | |

| | | | | | | |
|---|---|---|---|---|---|---|
| 1-year mortality rate mean<br>(s.d.)<br>median | 0.05<br>(0.01)<br>0.03 | 0.08<br>(0.003)<br>0.07 | 0.20<br>(0.03)<br>0.16 | 0.22<br>(0.02)<br>0.19 | 0.31<br>(0.03)<br>0.29 | |
| 1-year survival probability<br>Mean<br>(s.d.)<br>median | 0.95<br>(0.01)<br>0.97 | 0.92<br>(0.003)<br>0.94 | 0.80<br>(0.0275)<br>0.84 | 0.78<br>(0.02)<br>0.81 | 0.69<br>(0.03)<br>0.71 | |

## 5. Supplementary Table S5.: Co-occurrence of diseases A and B: phi parameter (all, female, male)

| Disease code A | Disease code B | Phi | Phi female | Phi male |
|---|---|---|---|---|
| 1B10 | BA00 | 0.07 | NA | 0.08 |
| 5A10 | BA00 | 0.07 | NA | 0.10 |
| 5A11 | 5B80 | 0.03 | 0.14 | NA |
| 5A11 | 8B11 | NA | NA | 0.03 |
| 5A11 | BA00 | 0.14 | 0.03 | 0.17 |
| 5A11 | BA4Z | 0.08 | NA | 0.12 |
| 5A11 | BD1Z | 0.02 | 0.14 | NA |
| 5A11 | BE2Z | 0.03 | NA | 0.06 |
| 5A11 | CA22 | 0.02 | NA | 0.02 |
| 5A11 | GB61 | 0.07 | 0.17 | 0.03 |
| 5A11 | GB6Z | NA | NA | 0.02 |
| 5A11 | QB42 | 0.01 | NA | 0.04 |
| 5A14 | 5B80 | 0.06 | 0.07 | 0.05 |
| 5A14 | 8E4Z | 0.15 | NA | NA |
| 5A14 | BA00 | NA | 0.09 | NA |
| 5A14 | BE2Z | 0.11 | 0.11 | 0.10 |
| 5A14 | CB41 | 0.07 | NA | 0.13 |
| 5A14 | GB61 | 0.03 | NA | 0.02 |

| | | | | |
|---|---|---|---|---|
| 5A14 | GB6Z | 0.06 | NA | NA |
| 5B80 | 5A11 | 0.03 | 0.14 | NA |
| 5B80 | 5A14 | 0.06 | 0.07 | 0.05 |
| 5B80 | BA00 | 0.06 | 0.09 | 0.05 |
| 5B80 | BD1Z | 0.04 | NA | NA |
| 5B80 | GB61 | 0.09 | 0.15 | 0.06 |
| 6B60 | 8B11 | 0.41 | NA | 0.49 |
| 8B11 | 5A11 | NA | NA | 0.03 |
| 8B11 | 6B60 | 0.41 | NA | 0.49 |
| 8B11 | BA00 | 0.15 | 0.16 | 0.14 |
| 8B11 | MB53 | 0.26 | NA | NA |
| 8E4Z | 5A14 | 0.15 | NA | NA |
| 8E4Z | BA00 | 0.05 | NA | NA |
| BA00 | 1B10 | 0.07 | NA | 0.08 |
| BA00 | 5A10 | 0.07 | NA | 0.10 |
| BA00 | 5A11 | 0.14 | 0.03 | 0.17 |
| BA00 | 5A14 | NA | 0.09 | NA |
| BA00 | 5B80 | 0.06 | 0.09 | 0.05 |
| BA00 | 8B11 | 0.15 | 0.16 | 0.14 |
| BA00 | 8E4Z | 0.05 | NA | NA |
| BA00 | BA4Z | 0.04 | NA | 0.03 |
| BA00 | BA5Z | 0.07 | 0.18 | NA |
| BA00 | BD10 | NA | NA | 0.08 |
| BA00 | BE2Z | 0.07 | 0.19 | NA |
| BA00 | CA22 | 0.08 | 0.12 | 0.07 |
| BA00 | CA40 | NA | NA | 0.03 |
| BA00 | CB40 | 0.06 | NA | 0.04 |
| BA00 | DB9Z | 0.10 | NA | 0.13 |
| BA00 | GB60 | 0.05 | NA | NA |
| BA00 | GB61 | NA | 0.09 | NA |

| | | | | |
|---|---|---|---|---|
| BA00 | GB6Z | 0.04 | NA | 0.07 |
| BA00 | QB42 | NA | NA | 0.03 |
| BA4Z | 5A11 | 0.08 | NA | 0.12 |
| BA4Z | BA00 | 0.04 | NA | 0.03 |
| BA4Z | BC81 | 0.14 | NA | NA |
| BA4Z | GB61 | 0.08 | NA | 0.12 |
| BA5Z | BA00 | 0.07 | 0.18 | NA |
| BC81 | BA4Z | 0.14 | NA | NA |
| BC81 | BD10 | 0.20 | NA | NA |
| BC81 | BD1Z | 0.13 | NA | NA |
| BD10 | BA00 | NA | NA | 0.08 |
| BD10 | BC81 | 0.20 | NA | NA |
| BD1Z | 5A11 | 0.02 | 0.14 | NA |
| BD1Z | 5B80 | 0.04 | NA | NA |
| BD1Z | BC81 | 0.13 | NA | NA |
| BD1Z | CA22 | 0.14 | NA | NA |
| BE2Z | 5A11 | 0.03 | NA | 0.06 |
| BE2Z | 5A14 | 0.11 | 0.11 | 0.10 |
| BE2Z | BA00 | 0.07 | 0.19 | NA |
| BE2Z | CB40 | 0.23 | NA | 0.20 |
| BE2Z | GB61 | 0.05 | 0.23 | NA |
| CA22 | 5A11 | 0.02 | NA | 0.02 |
| CA22 | BA00 | 0.08 | 0.12 | 0.07 |
| CA22 | BD1Z | 0.14 | NA | NA |
| CA40 | BA00 | NA | NA | 0.03 |
| CA40 | CB41 | 0.45 | NA | 0.55 |
| CB40 | BA00 | 0.06 | NA | 0.04 |
| CB40 | BE2Z | 0.23 | NA | 0.20 |
| CB41 | 5A14 | 0.07 | NA | 0.13 |
| CB41 | CA40 | 0.45 | NA | 0.55 |

| | | | | |
|---|---|---|---|---|
| DB9Z | BA00 | 0.10 | NA | 0.13 |
| GB60 | BA00 | 0.05 | NA | NA |
| GB61 | 5A11 | 0.07 | 0.17 | 0.03 |
| GB61 | 5A14 | 0.03 | NA | 0.02 |
| GB61 | 5B80 | 0.09 | 0.15 | 0.06 |
| GB61 | BA00 | NA | 0.09 | NA |
| GB61 | BA4Z | 0.08 | NA | 0.12 |
| GB61 | BE2Z | 0.05 | 0.23 | NA |
| GB61 | QB42 | 0.30 | NA | 0.38 |
| GB6Z | 5A11 | NA | NA | 0.02 |
| GB6Z | 5A14 | 0.06 | NA | NA |
| GB6Z | BA00 | 0.04 | NA | 0.07 |
| GB6Z | QB42 | 0.47 | 0.59 | 0.40 |
| MB53 | 8B11 | 0.26 | NA | NA |
| QB42 | 5A11 | 0.01 | NA | 0.04 |
| QB42 | BA00 | NA | NA | 0.03 |
| QB42 | GB61 | 0.30 | NA | 0.38 |
| QB42 | GB6Z | 0.47 | 0.59 | 0.40 |

6. Supplementary Table S6 -part 1. Spatial distribution of diseases as counts and relative frequency (prop)

| County | Counts | Prop | BA00 | BA00 prop | 5B80 | 5B80 prop | 5A14 | 5A14 prop | 5A10 | 5A10 prop | 5A11 | 5A11 prop | BE2Z | BE2Z prop | BC81 | BC8 prop |
|---|---|---|---|---|---|---|---|---|---|---|---|---|---|---|---|---|
| AB | 4 | 0.01 | 3 | 0.02 | 2 | 0.04 | 0 | 0 | 1 | 0.08 | 1 | 0.01 | 0 | 0 | 0 | 0 |
| AG | 5 | 0.01 | 2 | 0.01 | 0 | 0 | 0 | 0 | 0 | 0 | 1 | 0.01 | 0 | 0 | 1 | 0.04 |
| AR | 44 | 0.10 | 20 | 0.12 | 8 | 0.15 | 2 | 0.03 | 0 | 0 | 12 | 0.16 | 0 | 0 | 4 | 0.16 |
| B | 32 | 0.07 | 14 | 0.09 | 3 | 0.06 | 5 | 0.07 | 0 | 0 | 6 | 0.08 | 9 | 0.14 | 2 | 0.08 |
| BC | 9 | 0.02 | 4 | 0.03 | 1 | 0.02 | 1 | 0.02 | 0 | 0 | 2 | 0.03 | 0 | 0 | 3 | 0.12 |
| BH | 4 | 0.01 | 3 | 0.02 | 0 | 0 | 1 | 0.02 | 0 | 0 | 1 | 0.01 | 1 | 0.02 | 1 | 0.04 |

| | | | | | | | | | | | | | | | |
|---|---|---|---|---|---|---|---|---|---|---|---|---|---|---|---|
| BN | 10 | 0.02 | 7 | 0.04 | 0 | 0 | 0 | 0 | 2 | 0.17 | 4 | 0.05 | 1 | 0.02 | 2 | 0.08 |
| BT | 17 | 0.04 | 4 | 0.03 | 1 | 0.02 | 4 | 0.06 | 0 | 0 | 7 | 0.09 | 6 | 0.09 | 0 | 0 |
| BV | 12 | 0.03 | 2 | 0.01 | 3 | 0.07 | 0 | 0 | 1 | 0.08 | 6 | 0.08 | 0 | 0 | 0 | 0 |
| CJ | 4 | 0.01 | 2 | 0.01 | 0 | 0 | 3 | 0.05 | 0 | 0 | 0 | 0 | 1 | 0.02 | 0 | 0 |
| CS | 2 | 0.05 | 1 | 0.01 | 1 | 0.02 | 0 | 0 | 0 | 0 | 0 | 0 | 1 | 0.02 | 0 | 0 |
| CT | 8 | 0.02 | 4 | 0.03 | 2 | 0.04 | 3 | 0.05 | 0 | 0 | 1 | 0.01 | 1 | 0.02 | 0 | 0 |
| CV | 4 | 0.01 | 0 | 0 | 1 | 0.02 | 0 | 0 | 0 | 0 | 0 | 0 | 0 | 0 | 0 | 0 |
| DB | 4 | 0.01 | 1 | 0.01 | 0 | 0 | 0 | 0 | 1 | 0.08 | 0 | 0 | 0 | 0 | 0 | 0 |
| DJ | 1 | 0.01 | 0 | 0 | 1 | 0.02 | 1 | 0.02 | 1 | 0.08 | 0 | 0 | 0 | 0 | 0 | 0 |
| GJ | 2 | 0.05 | 0 | 0 | 1 | 0.02 | 0 | 0 | 0 | 0 | 0 | 0 | 0 | 0 | 0 | 0 |
| GL | 23 | 0.05 | 8 | 0.05 | 2 | 0.04 | 6 | 0.09 | 0 | 0 | 2 | 0.03 | 2 | 0.03 | 2 | 0.08 |
| GR | 3 | 0.01 | 1 | 0.01 | 0 | 0 | 0 | 0 | 0 | 0 | 1 | 0.01 | 0 | 0 | 0 | 0 |
| HN | 41 | 0.09 | 14 | 0.09 | 1 | 0.02 | 4 | 0.06 | 0 | 0 | 1 | 0.01 | 2 | 0.03 | 3 | 0.12 |
| IF | 8 | 0.02 | 2 | 0.01 | 2 | 0.04 | 5 | 0.08 | 0 | 0 | 2 | 0.03 | 2 | 0.03 | 0 | 0 |
| IL | 8 | 0.02 | 5 | 0.03 | 2 | 0.04 | 1 | 0.015 | 1 | 0.08 | 2 | 0.03 | 1 | 0.02 | 0 | 0 |
| IS | 7 | 0.02 | 4 | 0.03 | 0 | 0 | 2 | 0.03 | 0 | 0 | 0 | 0 | 0 | 0 | 0 | 0 |
| MH | 7 | 0.02 | 3 | 0.02 | 2 | 0.04 | 1 | 0.02 | 0 | 0 | 2 | 0.03 | 1 | 0.02 | 0 | 0 |
| MM | 1 | 0.01 | 0 | 0 | 0 | 0 | 0 | 0 | 0 | 0 | 0 | 0 | 0 | 0 | 0 | 0 |
| MS | 23 | 0.05 | 10 | 0.06 | 8 | 0.15 | 6 | 0.09 | 0 | 0 | 3 | 0.04 | 9 | 0.14 | 0 | 0 |
| NT | 17 | 0.04 | 3 | 0.02 | 1 | 0.02 | 3 | 0.05 | 0 | 0 | 1 | 0.01 | 0 | 0 | 0 | 0 |
| PH | 1 | 0.01 | 0 | 0 | 0 | 0 | 0 | 0 | 0 | 0 | 0 | 0 | 0 | 0 | 0 | 0 |
| SB | 14 | 0.03 | 7 | 0.04 | 1 | 0.02 | 1 | 0.02 | 0 | 0 | 0 | 0 | 7 | 0.11 | 0 | 0 |
| SJ | 3 | 0.01 | 2 | 0.01 | 0 | 0 | 0 | 0 | 0 | 0 | 1 | 0.01 | 0 | 0 | 0 | 0 |
| SM | 5 | 0.01 | 1 | 0.01 | 0 | 0 | 1 | 0.02 | 1 | 0.08 | 0 | 0 | 0 | 0 | 0 | 0 |
| SV | 67 | 0.16 | 18 | 0.11 | 4 | 0.08 | 7 | 0.11 | 1 | 0.08 | 8 | 0.11 | 12 | 0.18 | 5 | 0.2 |
| TL | 3 | 0.01 | 1 | 0.01 | 1 | 0.02 | 1 | 0.02 | 0 | 0 | 1 | 0.01 | 1 | 0.02 | 0 | 0 |
| TM | 24 | 0.06 | 12 | 0.07 | 3 | 0.06 | 4 | 0.06 | 2 | 0.17 | 7 | 0.09 | 4 | 0.06 | 1 | 0.04 |
| VL | 1 | 0.01 | 0 | 0 | 1 | 0.02 | 0 | 0 | 1 | 0.08 | 0 | 0 | 0 | 0 | 0 | 0 |
| VN | 12 | 0.03 | 4 | 0.03 | 0 | 0 | 3 | 0.05 | 0 | 0 | 3 | 0.04 | 5 | 0.08 | 1 | 0.04 |
| VS | 2 | 0.01 | 0 | 0 | 1 | 0.02 | 1 | 0.02 | 0 | 0 | 0 | 0 | 0 | 0 | 0 | 0 |

6. Supplementary Table S6 -part 2. Spatial distribution of diseases as counts and relative frequency

| BC81 | BC81prop | BD1Z | BD1Zprop | 8B11 | 8B11prop | GB61 | GB61prop | QB42 | QB42prop | GB6Z | GB6Zprop | CB41 | CB41prop | CA22 | CA22prop | FC | FCprop |
|---|---|---|---|---|---|---|---|---|---|---|---|---|---|---|---|---|---|
| 0 | 0 | 0 | 0 | 0 | 0 | 2 | 0.05 | 1 | 0.02 | 0 | 0 | 2 | 0.11 | 0 | 0 | 0 | 0 |
| 1 | 0.04 | 2 | 0.07 | 1 | 0.04 | 1 | 0.02 | 1 | 0.02 | 0 | 0 | 0 | 0 | 1 | 0.04 | 0 | 0 |
| 4 | 0.16 | 4 | 0.14 | 1 | 0.04 | 2 | 0.05 | 3 | 0.07 | 2 | 0.08 | 4 | 0.211 | 2 | 0.08 | 1 | 0.08 |
| 2 | 0.08 | 2 | 0.07 | 0 | 0 | 8 | 0.186 | 11 | 0.26 | 5 | 0.19 | 0 | 0 | 1 | 0.04 | 0 | 0 |
| 3 | 0.12 | 2 | 0.07 | 0 | 0 | 0 | 0 | 0 | 0 | 0 | 0 | 2 | 0.105 | 1 | 0.04 | 0 | 0 |
| 1 | 0.04 | 0 | 0 | 0 | 0 | 0 | 0 | 0 | 0 | 0 | 0 | 1 | 0.05 | 1 | 0.04 | 0 | 0 |
| 2 | 0.08 | 0 | 0 | 1 | 0.036 | 1 | 0.023 | 0 | 0 | 0 | 0 | 1 | 0.053 | 2 | 0.08 | 0 | 0 |
| 0 | 0 | 1 | 0.03 | 1 | 0.036 | 1 | 0.023 | 1 | 0.023 | 0 | 0 | 0 | 0 | 1 | 0.04 | 0 | 0 |
| 0 | 0 | 2 | 0.07 | 0 | 0 | 0 | 0 | 0 | 0 | 0 | 0 | 2 | 0.11 | 1 | 0.04 | 1 | 0.08 |
| 0 | 0 | 1 | 0.03 | 0 | 0 | 0 | 0 | 0 | 0 | 0 | 0 | 0 | 0 | 0 | 0 | 0 | 0 |
| 0 | 0 | 0 | 0 | 0 | 0 | 0 | 0 | 0 | 0 | 0 | 0 | 0 | 0 | 1 | 0.04 | 0 | 0 |
| 0 | 0 | 0 | 0 | 1 | 0.04 | 0 | 0 | 0 | 0 | 1 | 0.04 | 1 | 0.05 | 0 | 0 | 0 | 0 |
| 0 | 0 | 2 | 0.07 | 0 | 0 | 0 | 0 | 0 | 0 | 0 | 0 | 1 | 0.05 | 0 | 0 | 0 | 0 |
| 0 | 0 | 1 | 0.03 | 1 | 0.04 | 0 | 0 | 3 | 0.07 | 2 | 0.08 | 0 | 0 | 0 | 0 | 0 | 0 |
| 0 | 0 | 0 | 0 | 0 | 0 | 0 | 0 | 0 | 0 | 0 | 0 | 0 | 0 | 0 | 0 | 0 | 0 |
| 0 | 0 | 0 | 0 | 0 | 0 | 0 | 0 | 0 | 0 | 0 | 0 | 0 | 0 | 1 | 0.04 | 0 | 0 |
| 2 | 0.08 | 1 | 0.03 | 3 | 0.11 | 2 | 0.05 | 1 | 0.02 | 1 | 0.04 | 0 | 0 | 1 | 0.04 | 0 | 0 |
| 0 | 0 | 0 | 0 | 0 | 0 | 1 | 0.02 | 1 | 0.023 | 0 | 0 | 0 | 0 | 0 | 0 | 0 | 0 |
| 3 | 0.12 | 4 | 0.14 | 8 | 0.29 | 0 | 0 | 0 | 0 | 1 | 0.04 | 1 | 0.05 | 5 | 0.21 | 2 | 0.15 |
| 0 | 0 | 0 | 0 | 1 | 0.04 | 4 | 0.09 | 1 | 0.02 | 1 | 0.04 | 0 | 0 | 0 | 0 | 0 | 0 |

| | | | | | | | | | | | | | | | | | |
|---|---|---|---|---|---|---|---|---|---|---|---|---|---|---|---|---|---|
| 0 | 0 | 0 | 0 | 0 | 0 | 1 | 0.02 | 1 | 0.02 | 2 | 0.08 | 0 | 0 | 0 | 0 | 1 | 0.08 |
| 0 | 0 | 1 | 0.03 | 0 | 0 | 1 | 0.02 | 0 | 0 | 0 | 0 | 1 | 0.05 | 1 | 0.04 | 0 | 0 |
| 0 | 0 | 0 | 0 | 0 | 0 | 1 | 0.02 | 1 | 0.02 | 1 | 0.04 | 1 | 0.05 | 0 | 0 | 1 | 0.08 |
| 0 | 0 | 0 | 0 | 0 | 0 | 0 | 0 | 0 | 0 | 0 | 0 | 0 | 0 | 0 | 0 | 0 | 0 |
| 0 | 0 | 0 | 0 | 0 | 0 | 2 | 0.05 | 1 | 0.023 | 3 | 0.12 | 0 | 0 | 1 | 0.04 | 0 | 0 |
| 0 | 0 | 0 | 0 | 1 | 0.04 | 2 | 0.05 | 3 | 0.07 | 0 | 0 | 1 | 0.05 | 0 | 0 | 1 | 0.08 |
| 0 | 0 | 0 | 0 | 0 | 0 | 0 | 0 | 0 | 0 | 0 | 0 | 0 | 0 | 0 | 0 | 0 | 0 |
| 0 | 0 | 0 | 0 | 0 | 0 | 3 | 0.07 | 1 | 0.02 | 1 | 0.04 | 0 | 0 | 0 | 0 | 1 | 0.08 |
| 0 | 0 | 0 | 0 | 1 | 0.036 | 0 | 0 | 0 | 0 | 0 | 0 | 0 | 0 | 0 | 0 | 0 | 0 |
| 0 | 0 | 0 | 0 | 0 | 0 | 0 | 0 | 1 | 0.02 | 1 | 0.04 | 0 | 0 | 0 | 0 | 0 | 0 |
| 5 | 0.2 | 3 | 0.10 | 7 | 0.25 | 7 | 0.16 | 7 | 0.16 | 1 | 0.04 | 0 | 0 | 3 | 0.13 | 5 | 0.39 |
| 0 | 0 | 0 | 0 | 0 | 0 | 1 | 0.02 | 0 | 0 | 0 | 0 | 0 | 0 | 0 | 0 | 0 | 0 |
| 1 | 0.04 | 1 | 0.03 | 1 | 0.04 | 2 | 0.05 | 2 | 0.05 | 2 | 0.08 | 1 | 0.05 | 1 | 0.04 | 0 | 0 |
| 0 | 0 | 0 | 0 | 0 | 0 | 0 | 0 | 0 | 0 | 0 | 0 | 0 | 0 | 0 | 0 | 0 | 0 |
| 1 | 0.04 | 1 | 0.03 | 0 | 0 | 1 | 0.023 | 3 | 0.07 | 2 | 0.08 | 0 | 0 | 0 | 0 | 0 | 0 |
| 0 | 0 | 1 | 0.03 | 0 | 0 | 0 | 0 | 0 | 0 | 0 | 0 | 0 | 0 | 0 | 0 | 0 | 0 |

Legend Supplementary Table S6

8B11 Cerebral ischaemic stroke

BA5Z Chronic ischaemic heart disease, unspecified

MB53 Hemiplegia

QB42 Dependence on renal dialysis

BA00 Essential hypertension

GB61 Chronic kidney disease

6B60 Dissociative neurological symptom disorder

GB60 Acute kidney failure

5A10 Diabetes mellitus type 1

CA40 Pneumonia

1B10 Tuberculosis of the respiratory system

CB41 Respiratory failure

5B80 Overweight or localised adiposity

GB6Z Kidney failure, unspecified

BD1Z Heart failure, unspecified

CB40 Certain diseases of the respiratory system

CA22 Chronic obstructive pulmonary disease

BE2Z Diseases of the circulatory system, unspecified

8E4Z Other disorders of the nervous system, unspecified

BA4Z Acute ischaemic heart disease, unspecified

BC81 Supraventricular tachyarrhythmia

5A11 Diabetes mellitus type 2

BD10 Congestive heart failure

DB9Z Diseases of liver, unspecified

5A14 Diabetes mellitus, type unspecified

7. Supplementary Table S7 -part 1. Spatial distribution of aggregated groups of diseases as counts and relative frequency (prop)

| County | Counts | Prop | 11 | 11 prop | 5 | 5 prop | 24 | 24 prop | 16 | 16 prop | 2 | 2 prop | 12 | 12 prop | 21 | 21 prop | 1 | 1 prop |
|---|---|---|---|---|---|---|---|---|---|---|---|---|---|---|---|---|---|---|
| AB | 4 | 0.01 | 4 | 0.02 | 3 | 0.02 | 1 | 0.02 | 2 | 0.03 | 0 | 0 | 2 | 0.03 | 0 | 0 | 2 | 0.1 |
| AG | 5 | 0.01 | 4 | 0.02 | 1 | 0.01 | 1 | 0.02 | 1 | 0.01 | 0 | 0 | 2 | 0.03 | 2 | 0.08 | 0 | 0 |
| AR | 44 | 0.10 | 26 | 0.10 | 19 | 0.10 | 3 | 0.06 | 5 | 0.06 | 4 | 0.1 | 10 | 0.15 | 4 | 0.17 | 2 | 0.1 |
| B | 32 | 0.07 | 21 | 0.08 | 14 | 0.08 | 13 | 0.241 | 13 | 0.165 | 4 | 0.1 | 4 | 0.06 | 0 | 0 | 2 | 0.1 |
| BC | 9 | 0.02 | 6 | 0.02 | 4 | 0.02 | 0 | 0 | 3 | 0.04 | 1 | 0.03 | 2 | 0.03 | 0 | 0 | 0 | 0 |
| BH | 4 | 0.01 | 3 | 0.01 | 2 | 0.01 | 0 | 0 | 0 | 0 | 0 | 0 | 2 | 0.03 | 1 | 0.04 | 0 | 0 |
| BN | 10 | 0.02 | 9 | 0.04 | 6 | 0.03 | 0 | 0 | 2 | 0.03 | 1 | 0.03 | 4 | 0.06 | 1 | 0.04 | 0 | 0 |
| BT | 17 | 0.04 | 8 | 0.03 | 12 | 0.07 | 1 | 0.02 | 2 | 0.03 | 3 | 0.08 | 2 | 0.03 | 1 | 0.04 | 0 | 0 |
| BV | 12 | 0.03 | 7 | 0.03 | 8 | 0.04 | 0 | 0 | 0 | 0 | 1 | 0.03 | 2 | 0.03 | 1 | 0.04 | 1 | 0.05 |
| CJ | 4 | 0.01 | 4 | 0.02 | 3 | 0.02 | 0 | 0 | 1 | 0.01 | 1 | 0.03 | 1 | 0.02 | 0 | 0 | 1 | 0.05 |
| CS | 2 | 0.01 | 2 | 0.01 | 1 | 0.01 | 1 | 0.02 | 0 | 0 | 0 | 0 | 1 | 0.02 | 0 | 0 | 1 | 0.05 |
| CT | 8 | 0.02 | 4 | 0.02 | 6 | 0.03 | 1 | 0.02 | 1 | 0.01 | 0 | 0 | 2 | 0.03 | 1 | 0.04 | 1 | 0.05 |
| CV | 4 | 0.01 | 2 | 0.01 | 1 | 0.01 | 1 | 0.02 | 0 | 0 | 0 | 0 | 1 | 0.02 | 1 | 0.04 | 0 | 0 |
| DB | 4 | 0.01 | 2 | 0.01 | 1 | 0.01 | 3 | 0.06 | 2 | 0.03 | 0 | 0 | 0 | 0 | 0 | 0 | 2 | 0.1 |
| DJ | 1 | 0.01 | 0 | 0 | 1 | 0.01 | 0 | 0 | 0 | 0 | 0 | 0 | 0 | 0 | 0 | 0 | 0 | 0 |
| GJ | 2 | 0.01 | 1 | 0.01 | 1 | 0.01 | 0 | 0 | 0 | 0 | 0 | 0 | 1 | 0.02 | 1 | 0.04 | 0 | 0 |
| GL | 23 | 0.05 | 13 | 0. | 10 | 0.05 | 1 | 0.02 | 3 | 0.04 | 0 | 0 | 1 | 0.02 | 1 | 0.04 | 1 | 0.05 |
| GR | 3 | 0.01 | 2 | 0.01 | 1 | 0.01 | 1 | 0.02 | 1 | 0.01 | 0 | 0 | 0 | 0 | 0 | 0 | 0 | 0 |
| HN | 41 | 0.09 | 22 | 0.09 | 6 | 0.03 | 1 | 0.02 | 2 | 0.03 | 12 | 0.3 | 6 | 0.09 | 4 | 0.17 | 0 | 0 |
| IF | 8 | 0.02 | 4 | 0.02 | 7 | 0.05 | 1 | 0.02 | 5 | 0.06 | 1 | 0.03 | 0 | 0 | 0 | 0 | 0 | 0 |
| IL | 8 | 0.02 | 5 | 0.02 | 6 | 0.03 | 1 | 0.02 | 4 | 0.05 | 0 | 0 | 0 | 0 | 0 | 0 | 1 | 0.05 |
| IS | 7 | 0.02 | 4 | 0.02 | 2 | 0.01 | 0 | 0 | 1 | 0.01 | 1 | 0.03 | 1 | 0.02 | 0 | 0 | 1 | 0.05 |
| MH | 7 | 0.02 | 3 | 0.01 | 4 | 0.02 | 1 | 0.02 | 3 | 0.04 | 0 | 0 | 2 | 0.03 | 0 | 0 | 2 | 0.1 |
| MM | 1 | 0.01 | 0 | 0 | 0 | 0 | 0 | 0 | 0 | 0 | 0 | 0 | 0 | 0 | 0 | 0 | 0 | 0 |
| MS | 23 | 0.05 | 14 | 0.06 | 14 | 0.08 | 1 | 0.02 | 5 | 0.06 | 0 | 0 | 2 | 0.03 | 0 | 0 | 0 | 0 |

| | | | | | | | | | | | | | | | | | | |
|---|---|---|---|---|---|---|---|---|---|---|---|---|---|---|---|---|---|---|
| NT | 17 | 0.04 | 5 | 0.02 | 4 | 0.02 | 3 | 0.06 | 2 | 0.03 | 4 | 0.1 | 1 | 0.02 | 1 | 0.04 | 0 | 0 |
| PH | 1 | 0.01 | 0 | 0 | 0 | 0 | 0 | 0 | 0 | 0 | 0 | 0 | 0 | 0 | 0 | 0 | 0 | 0 |
| SB | 14 | 0.03 | 11 | 0.04 | 2 | 0.01 | 2 | 0.04 | 4 | 0.05 | 1 | 0.03 | 1 | 0.02 | 0 | 0 | 1 | 0.05 |
| SJ | 3 | 0.01 | 2 | 0.01 | 1 | 0.01 | 0 | 0 | 0 | 0 | 0 | 0 | 2 | 0.03 | 0 | 0 | 0 | 0 |
| SM | 5 | 0.01 | 2 | 0.01 | 2 | 0.01 | 1 | 0.02 | 1 | 0.01 | 1 | 0.03 | 0 | 0 | 1 | 0.04 | 0 | 0 |
| SV | 67 | 0.16 | 36 | 0.14 | 19 | 0.10 | 10 | 0.19 | 8 | 0.101 | 3 | 0.075 | 8 | 0.12 | 4 | 0.17 | 1 | 0.05 |
| TL | 3 | 0.01 | 2 | 0.01 | 2 | 0.01 | 0 | 0 | 1 | 0.01 | 0 | 0 | 0 | 0 | 0 | 0 | 0 | 0 |
| TM | 24 | 0.06 | 18 | 0.07 | 14 | 0.08 | 2 | 0.04 | 4 | 0.05 | 2 | 0.05 | 4 | 0.06 | 0 | 0 | 1 | 0.05 |
| VL | 1 | 0.01 | 0 | 0 | 1 | 0.01 | 0 | 0 | 0 | 0 | 0 | 0 | 0 | 0 | 0 | 0 | 0 | 0 |
| VN | 12 | 0.03 | 9 | 0.04 | 6 | 0.03 | 3 | 0.06 | 3 | 0.04 | 0 | 0 | 3 | 0.05 | 0 | 0 | 0 | 0 |
| VS | 2 | 0.01 | 1 | 0.01 | 1 | 0.01 | 1 | 0.02 | 0 | 0 | 0 | 0 | 0 | 0 | 0 | 0 | 0 | 0 |

7. Supplementary Table S7 -part 2. Spatial distribution of aggregated groups of diseases as counts and relative frequency (prop)

| County | 6 | 6 prop | 8 | 8 prop | 13 | 13 prop | 0 | 0 prop |
|---|---|---|---|---|---|---|---|---|
| AB | 0 | 0 | 0 | 0 | 0 | 0 | 0 | 0 |
| AG | 1 | 0.03 | 1 | 0.02 | 2 | 0.05 | 0 | 0 |
| AR | 1 | 0.03 | 4 | 0.07 | 3 | 0.07 | 1 | 0.08 |
| B | 2 | 0.06 | 4 | 0.07 | 1 | 0.02 | 0 | 0 |
| BC | 1 | 0.03 | 0 | 0 | 1 | 0.02 | 0 | 0 |
| BH | 0 | 0 | 1 | 0.02 | 0 | 0 | 0 | 0 |
| BN | 1 | 0.03 | 1 | 0.02 | 0 | 0 | 0 | 0 |
| BT | 0 | 0 | 2 | 0.03 | 0 | 0 | 0 | 0 |
| BV | 2 | 0.06 | 2 | 0.03 | 4 | 0.09 | 1 | 0.08 |
| CJ | 1 | 0.03 | 1 | 0.02 | 2 | 0.05 | 0 | 0 |
| CS | 1 | 0.03 | 0 | 0 | 0 | 0 | 0 | 0 |
| CT | 0 | 0 | 1 | 0.02 | 1 | 0.02 | 0 | 0 |
| CV | 0 | 0 | 0 | 0 | 0 | 0 | 0 | 0 |

| | | | | | | | | |
|---|---|---|---|---|---|---|---|---|
| DB | 0 | 0 | 1 | 0.02 | 1 | 0.02 | 0 | 0 |
| DJ | 0 | 0 | 0 | 0 | 0 | 0 | 0 | 0 |
| GJ | 0 | 0 | 0 | 0 | 0 | 0 | 0 | 0 |
| GL | 7 | 0.19 | 8 | 0.13 | 2 | 0.05 | 0 | 0 |
| GR | 0 | 0 | 0 | 0 | 1 | 0.02 | 0 | 0 |
| HN | 6 | 0.17 | 9 | 0.15 | 3 | 0.07 | 2 | 0.15 |
| IF | 0 | 0 | 3 | 0.05 | 1 | 0.02 | 0 | 0 |
| IL | 0 | 0 | 0 | 0 | 0 | 0 | 1 | 0.08 |
| IS | 1 | 0.03 | 0 | 0 | 2 | 0.05 | 0 | 0 |
| MH | 0 | 0 | 1 | 0.02 | 2 | 0.05 | 1 | 0.08 |
| MM | 0 | 0 | 1 | 0.02 | 0 | 0 | 0 | 0 |
| MS | 1 | 0.03 | 1 | 0.02 | 4 | 0.09 | 0 | 0 |
| NT | 0 | 0 | 2 | 0.03 | 4 | 0.09 | 1 | 0.08 |
| PH | 0 | 0 | 0 | 0 | 1 | 0.02 | 0 | 0 |
| SB | 0 | 0 | 3 | 0.05 | 2 | 0.05 | 1 | 0.08 |
| SJ | 1 | 0.03 | 1 | 0.02 | 0 | 0 | 0 | 0 |
| SM | 1 | 0.03 | 0 | 0 | 0 | 0 | 0 | 0 |
| SV | 7 | 0.19 | 11 | 0.18 | 3 | 0.07 | 5 | 0.39 |
| TL | 0 | 0 | 0 | 0 | 0 | 0 | 0 | 0 |
| TM | 1 | 0.03 | 2 | 0.03 | 2 | 0.05 | 0 | 0 |
| VL | 0 | 0 | 0 | 0 | 0 | 0 | 0 | 0 |
| VN | 1 | 0.03 | 1 | 0.02 | 2 | 0.05 | 0 | 0 |
| VS | 0 | 0 | 0 | 0 | 0 | 0 | 0 | 0 |

Legend Supplementary Table S7

| | |
|---|---|
| 0 | No comorbidities |
| 11 | Diseases of the circulatory system |
| 5 | Endocrine, nutritional or metabolic diseases |
| 16 | Diseases of the genitourinary system |
| 12 | Diseases of the respiratory system |
| 8 | Diseases of the nervous system |
| 13 | Diseases of the digestive system |
| 2 | Neoplasms |
| 6 | Mental, behavioural or neurodevelopmental disorders |
| 24 | Factors influencing health status or contact with health services |
| 21 | Symptoms, signs or clinical findings, not elsewhere classified |
| 1 | Certain infectious or parasitic disease |

## 8. Section 8: Death risk and survival probability

Starting from the 1-year crude death risk for the general Romanian population in 2020 calculated by the United Nations [1], we estimated the Romanian gender-specific baseline crude death rates as 89 (female) respectively 177 (male) per 1000 people by considering the gender ratio of the death rates from the World Bank statistics for 2017 [2]. Hence, we overestimated the death rate for the group under 50 and at the baseline (50), and we retrospectively computed the point prediction of the individual death risk for each patient from our study. The 1-year survival probability is one minus the death rate. The empirical mean, median and standard deviation of both risks were computed starting from a beta model for the distribution of probabilities. [3,4] The forecast quality and accuracy of extrapolation for a Romanian study need to be further investigated.[3]

References


1. Macrotrends (https://www.macrotrends.net/countries/ROU/ ).
2. Trading Economics (https://tradingeconomics.com/romania/death-rate-crude-per-1-000-people-wb-data.html).
3. Henderson, R., Keiding, N. Individual survival time prediction using statistical models. *Journal of medical ethics* 31,703-6 (2006).
4. Ferrari, S., Cribari-Neto, F. Beta Regression for Modelling Rates and Proportions. *Journal of Applied Statistics* 31(7), 799-815 (2004).
